# Application and accuracy analysis of three-dimensional transvaginal ultrasound in the diagnosis of endometrial lesions in postmenopausal women

**DOI:** 10.12669/pjms.38.5.5331

**Published:** 2022

**Authors:** Liu Li, Qian Chen

**Affiliations:** 1 Liu Li, Department of Ultrasonography, Tian Jin Central Hospital of Gynecology and Obstetrics, Tianjin 300052, China; 2 Qian Chen, Department of Ultrasonography, Tian Jin Central Hospital of Gynecology and Obstetrics, Tianjin 300052, China

**Keywords:** Menopause, Endometrial lesion, Three-dimensional transvaginal ultrasound, Endometrial thickness, Micro-vessel density

## Abstract

**Objectives:**

To evaluate the value of three-dimensional transvaginal ultrasound (3D-TVS) for endometrial lesions in postmenopausal women, so as to improve the accuracy of clinical diagnosis of endometrial lesions in postmenopausal women.

**Methods:**

A total of 170 female patients admitted to Tianjin Central Hospital of Gynecology and Obstetrics from April 2018 to April 2020 were included in this study. Among them, 110 patients with pathologically confirmed postmenopausal endometrial lesions were divided into the study group (65 patients with benign lesions, 45 patients with malignant lesions), while 60 healthy postmenopausal women were divided into the volunteer group. PASS software was used to calculate the sample size. 3D-TVS was performed in both groups, and the results of the different groups were compared and analyzed.

**Results:**

The pathological results were invoked as the gold standard for detection, and the accuracy, sensitivity and specificity of 3D-TVS were 97.27%, 77.78% and 80% respectively. Patients in the study group had higher endometrial thickness and endometrial volume than those in the volunteer group (p<0.05). The results of ultrasound parameters in the benign lesion group were lower than those in the malignant lesion group (p<0.05), while the vascular index and micro-vessel density in the malignant lesion group were significantly higher than those in the benign lesion group (p<0.05).

**Conclusion:**

3D-TVS has obvious clinical value in the diagnosis of endometrial lesions in postmenopausal women, boasting a variety of advantages, such as high accuracy, sensitivity and specificity, effectively reflecting the blood flow of patients with endometrial lesions, and further determining the degree of benign and malignant lesions in patients.

## INTRODUCTION

Postmenopausal vaginal bleeding in women is clinically extremely frequent, with relatively complicated causes. Hysteroscopy and curettage (staged diagnostic curettage) are the traditional approaches for clinical diagnosis of vaginal bleeding. Despite the high diagnostic value, both approaches are only performed during invasive examination, which may cause physical damage to patients, resulting in intrauterine infection, perforation or other injuries.^1^

In the wake of the development of clinical ultrasound diagnostic technology, ultrasound has gradually become the principal clinical diagnostic tool for postmenopausal vaginal bleeding by virtue of its advantages of non-invasiveness and ease of operation. It can also be used to observe the blood flow in the uterus and the location of the lesion more intuitively, while the unit Doppler ultrasound imaging technology can more intuitively display the small blood vessels in the uterus, thus improving the diagnostic accuracy.^2^ Our objective was to investigate for the accuracy of 3D-TVS in detecting endometrial lesions.

## METHODS

A total of 170 female patients admitted to Tianjin Central Hospital of Gynecology and Obstetrics from April 2018 to April 2020 were recruited for this study. Among them, 110 patients with pathologically confirmed postmenopausal endometrial lesions were divided into the study group, while 60 healthy postmenopausal women were divided into the volunteer group. Sixty patients in the volunteer group had an average age of (59.65±6.25) years, an average length of menopause of (9.15±6.25) years, and an average number of pregnancies of (2.57±1.11) years. 110 patients in the study group had an average age of (60.32±7.16) years, an average length of menopause of (9.21±6.62) years, and an average number of pregnancies of (2.86±1.28). Before the two groups of patients were studied, the results of individual baseline data were compared, p>0.05. Prior to study, the study content was explained to the patients and their guardians, and consent was obtained.

### Ethical Approval:

The study was approved by the Institutional Ethics Committee of Tian Jin Central Hospital of Gynecology and Obstetrics on March 18, 2018 (No.:2018020), and written informed consent was obtained from all participants

### Inclusion Criteria:

- Patients over 40 years of age and more than one year of menopause;
- Patients with endometrial lesions who had received 3D-TVS before surgery;
- Patients with endometrial lesions determined by surgical pathology;
- Patients who had not used hormone drugs and had no history of chemotherapy or radiotherapy in the first six months of the study;
- Patients without a history of endocrine diseases.

### Exclusion Criteria:

- Patients with organ lesions, such as renal failure;
- Patients with intrauterine device in the uterus;
- Patients with uterine fibroids or adenomyosis;
- Patients with endometrial inhomogeneous thickening less than 5mm by conventional two-dimensional ultrasound;^3^
- Patients who are unable to participate in the entire study.

### Instruments:

Ultrasonic diagnostic apparatus (model LOGIQ-E8) with vaginal probe frequency set at 7MHz was used for this study. Patients were examined at the vesical calculi position. Specifically, patients underwent conventional two-dimensional ultrasound, followed by a color gain setting under color Doppler to improve the detection rate of low-velocity blood flow. The color blood flow velocity waveform was depicted by pulse Doppler, and three new continuous cycles were selected for analysis. Finally, the results of various parameters were recorded, including pulsation index (PI), peak systolic velocity (PSV) and resistance index (RI).

After checking the probe and confirming no error, a power Doppler was activated with a range and a wall filter set. Low flow in small branches was displayed without background noise. In 3D mode, patients are required to hold their breath and remain still to receive 3D scanning, with the scanning position of endometrium and the scanning time of 3s-5s. The probe was rotated to save the 3D stereoscopic image of blood distribution in the target area.^4^

In ABC view, the acquired 3D stereoscopic image of blood distribution was played back, and the image was properly adjusted and enlarged by adjusting the contour of the endometrium was clearly displayed in the image. The ROI sampling frame was used to adjust the acquired image to ensure that the image could include endometrium and adjacent tissues at the same time, and only endometrial blood flow was included in the image. The dynamic rotation image could clearly display the direction and branch of blood vessels as well as the number of blood vessels.^5^

### Study Indexes:

With the help of VOCAL software, the endometrium volume and endometrium thickness were calculated, the multi-directional coordinate axis was adjusted, and the section was selected. In the process of volume measurement, the contour of section intima was scanned manually, and 3D reconstruction of intima morphology was automatically carried out, and the volume was calculated. After repeated measurement for three times, the mean value of measurement was taken.^6^

### Blood flow quantification:

Counting standard for the total number of endometrial blood vessels: If a single blood vessel does not carry any branches, it is a blood vessel; If a single blood vessel carries branches, it is calculated based on the number of branches of peripheral vessels.^7^ The total number of blood vessels was compared with the volume of the endometrium, and the result was the number of blood vessels per unit volume, which could be used as the vascular index (VI) that responds to the blood supply at the tumor site.^8^ The measurement was carried out 3 times and then the average value was taken.

### Micro-vessel density (MVD):

The focal tissue sections of patients were taken by surgery or curettage and stained by SP immunohistochemistry. CD34 method was adopted for vascular labeling. Phosphate buffer was selected for negative control and MVD was counted according to Weidner method.^9^ Methods: Three areas with the highest vascular density were selected under low magnification field, three micro-vascular areas were selected under high magnification field, and the average value was selected as the value of MVD. 4) The pathological results were invoked as the gold standard to calculate the accuracy, sensitivity and specificity of 3D-TVS.

### Data Processing:

All the data in the study were processed by the statistical software SPSS 21.0.1. Enumeration data were expressed as (%) and tested by x². Measurement data were expressed as (`x±s), and t test was adopted for intra-group pairing.

## RESULTS

According to the data in Table-I, the pathological results were invoked as the gold standard for detection, and the accuracy, sensitivity and specificity of 3D-TVS were 97.27%, 77.78% and 80% respectively compared with the pathological results.As shown in Table-II, patients in the study group had higher endometrial thickness and endometrial volume than those in the volunteer group (p<0.05). The results of ultrasound parameters in the benign lesion group were lower than those in the malignant lesion group (p<0.05), while the vascular index and MVD in the malignant lesion group were significantly higher than those in the benign lesion group (p<0.05). Table-III.

**Table I:** Questionnaire of 3D-TVS and pathological results.

3D-TVS diagnosis	Pathological diagnosis		Total

	Malignant lesions	Benign lesions	
Malignant lesions	35	13	48
Benign lesions	10	52	62

Total	45	65	110

**Table II:** Questionnaire for comparison of endometrial thickness and endometrial volume between two groups (`x±s).

Item	Endometrial thickness (mm)	Endometrial volume (cm^3^)
Study group (n=110)	12.62±6.25	7.95±4.01
Voluntary group (n=60)	3.12±1.15	0.79±0.58
t value	11.6517	13.7345
p value	0.000	0.000

**Table III:** Questionnaire for comparison of ultrasound parameters and MVD in patients with benign and malignant lesions (`x±s).

Item	PI	RI	PSV (cm/s)	VI (strip/cm^3^)	MVD (strip/HP)
Benign lesions (n=65)	0.62±0.04	0.58±0.04	8.17±0.42	0.32±1.58	10.25±2.68
Malignant lesions (n=45)	0.24±0.11	0.22±0.07	6.15±1.98	1.61±1.78	27.52±7.98
t value	25.9160	35.2337	8.0204	4.2049	16.3997
p value	0.000	0.000	0.000	0.000	0.000

## DISCUSSION

Postmenopausal women are usually accompanied by a series of adverse reactions, such as a gradual decline in ovarian function, a gradual decline in the level of stimulating hormones in the body, atrophy of reproductive organs, decreased immune function, and increased incidence of endometrial lesions. Patients with common endometrial lesions are mainly associated with endometrial polyps, or uterine cancer and other diseases. Clinically, most of these patients will have symptoms of vaginal bleeding, while a small number of patients have no obvious symptoms.^10^ The traditional methods for the detection of postmenopausal vaginal bleeding are predominantly invasive, which are not only complicated and harmful to patients, but even aggravate patients’ conditions. 3D-TVS, by contrast, has gained popularity in clinics because it is simple to operate and does no harm to patients. In order to improve the clinical efficacy of patients with postmenopausal endometrial lesions, emphasis should be given to improving the accuracy of clinical diagnosis and mastering the patients’ conditions.

Three-dimensional Doppler ultrasound diagnostic technology, a blood flow imaging technology based on the detection of intensity changes in the Doppler signal and the movement of red blood cells, is highly sensitive to low-velocity blood vessels and low-volume blood vessels in the human body. Meanwhile, such a technology can present stereoscopic images for visually observing the blood vessels and organ structures inside human masses, which can further enhance the accuracy of diagnosis.^11^,^12^ Three-dimensional Doppler ultrasound technology be used for analyzing blood flow quantification by virtue of blood flow histograms. In the study, compared with the pathological results, the accuracy, sensitivity and specificity of 3D-TVS were 97.27%, 77.78% and 80% respectively, indicating that 3D-TVS has certain clinical diagnostic accuracy for patients with postmenopausal endometrial lesions. Patients with endometrial lesions are mostly caused by the localized changes of the endometrium, with clear boundaries of lesions. With the severity of the disease, the basal layer of the endometrium of patients will appear varying degrees of infiltration, resulting in blurred edges, irregularities, and increasing volume. Therefore, patients with endometrial lesions have increased endometrial volume and thickness compared with healthy patients.^13^

According to the results, endometrial thickness and endometrial volume of patients in the study group were significantly higher than those in the volunteer group, (p<0.05); Both the growth and infiltration of cancer foci require more nutrients. Moreover, the endometrium and its basal layer of patients will also undergo certain changes. The emergence of new organisms leads to the continuous increase of the endometrium and uneven echo phenomenon in ultrasound.^14^ The blood in the tumor is mainly supplied from the endometrium and its basal layer. The growth of new blood vessels in the tumor is extremely fast, but the wall of new blood vessels lacks muscle layer, resulting in low resistance blood flow. The results of ultrasound parameters in the benign lesion group were lower than those in the malignant lesion group (p<0.05), while the vascular index and MVD in the malignant lesion group were significantly higher than those in the benign lesion group (p<0.05). It was also noted in the study that in 3D-TVS detection, patients are required to keep still and hold their breath. Once patients move, 3D images will be deformed, affecting the final detection and diagnosis results.^15^

### Limitations of the study:

The number of subjects included in this study is limited. In addition, we only analyzed the cases from our hospital, which may not be representative enough. We look forward to a multi-center study in the future to reach more comprehensive conclusions. The study had a small sample size, short follow-up period and no more detailed subgroup comparison. Our findings need to be further confirmed by more in-depth studies in the future.

## CONCLUSION

3D-TVS has obvious clinical value in the diagnosis of endometrial lesions in postmenopausal women, boasting a variety of advantages, such as high accuracy, sensitivity and specificity, effectively reflecting the blood flow of patients with endometrial lesions, and further determining the degree of benign and malignant lesions in patients.

### Authors’ Contributions:

**LL:** Designed this study, prepared this manuscript, are responsible and accountable for the accuracy and integrity of the work.

**QC:** Collected and analyzed clinical data.
